# Fatal Expanded Dengue Syndrome Mimicking Heat Stroke With Hyperpyrexia, Subarachnoid Hemorrhage, and Refractory Multiorgan Dysfunction: A Case Report

**DOI:** 10.7759/cureus.108202

**Published:** 2026-05-03

**Authors:** Kyaw Zin Aung, Ei Ei Cho, Su Su Htun, Naw Eh Law Saw, Thinn Thiri Soe, Lin Swe Htet

**Affiliations:** 1 Internal Medicine, Kulhudhuffushi Regional Hospital, Kulhudhuffushi City, MDV; 2 Emergency, Kulhudhuffushi Regional Hospital, Kulhudhuffushi City, MDV; 3 Internal Medicine, Kanditheemu Health Centre, Kanditheemu, MDV

**Keywords:** acute subarachnoid haemorrhage, critical care, expanded dengue syndrome, heat stroke mimic, multiorgan dysfunction syndrome

## Abstract

Dengue is a major mosquito-borne viral illness affecting tropical and subtropical regions worldwide and remains an important cause of critical care admissions. Although most infections are self-limited, a subset progresses to severe dengue characterized by plasma leakage, shock, severe bleeding, and end-organ dysfunction. Neurological manifestations are uncommon but increasingly recognized under the spectrum of expanded dengue syndrome, including seizures, encephalopathy, cerebral edema, ischemic stroke, and intracranial hemorrhage. We report a fatal case of a 29-year-old previously healthy man with significant occupational and environmental heat exposure in a tropical setting. He presented after two generalized tonic-clonic seizures followed by profound unconsciousness. He had experienced four days of continuous fever, retro-orbital pain, myalgia, and arthralgia and had been diagnosed one day earlier with NS1-positive dengue fever without warning signs. On arrival, his temperature was 41°C, and his skin was hot and dry; shortly afterward, he developed respiratory arrest requiring emergency intubation, making environmental heat stroke an important initial differential diagnosis. Brain imaging demonstrated subarachnoid hemorrhage and diffuse cerebral edema. During intensive care, he developed progressive hemoconcentration, dengue shock syndrome, coagulopathy suggestive of disseminated intravascular coagulation, acute kidney injury, hepatic dysfunction, lactic acidosis, and refractory shock despite fluid resuscitation, vasopressor support, neurocritical care, and blood product replacement, ultimately resulting in death. This case highlights the diagnostic challenge posed by the overlap between severe dengue and heat-stroke-like presentations, particularly in individuals with significant exposure to environmental heat.

## Introduction

Dengue is a rapidly expanding arboviral disease affecting tropical and subtropical regions worldwide, with the World Health Organization estimating 100-400 million infections annually [1]. Severe dengue is characterized by critical plasma leakage, shock, severe bleeding, or severe organ involvement and carries significant mortality if not recognized early [1].

Atypical or severe organ-specific manifestations are increasingly described under the term "expanded dengue syndrome," which includes neurological, hepatic, renal, cardiac, and hematological complications [2,3]. Neurological involvement occurs in approximately 1-5% of hospitalized cases and ranges from encephalopathy and seizures to intracranial hemorrhage and subarachnoid hemorrhage [3-5]. Spontaneous intracranial bleeding is uncommon and reflects multifactorial mechanisms, including thrombocytopenia, endothelial dysfunction, hepatic coagulopathy, disseminated intravascular coagulation, and vascular fragility [6-8].

In dengue-endemic tropical settings, severe dengue with extreme hyperpyrexia may resemble environmental heat stroke, particularly in individuals with prolonged occupational or environmental heat exposure. This diagnostic overlap may delay recognition of the plasma leakage phase and timely management, with potential adverse outcomes.

We report a fatal case of expanded dengue syndrome with early neurological involvement, subarachnoid hemorrhage, dengue shock syndrome, coagulopathy, and refractory multiorgan dysfunction, in which the initial presentation closely mimicked environmental heat stroke [2,9].

## Case presentation

A 29-year-old previously healthy man with significant occupational heat exposure was brought to the emergency department after two witnessed generalized tonic-clonic seizures at home, each lasting approximately one minute and associated with tongue biting and urinary and fecal incontinence. Following the seizures, he remained deeply unconscious.

According to family members, he had experienced four days of continuous high-grade fever associated with severe headache, retro-orbital pain, diffuse myalgia, and arthralgia, without a reported rash or neck stiffness. One day prior to admission, he had been evaluated by a general practitioner and diagnosed with dengue fever without warning signs based on positive NS1 antigen testing, leukopenia, and thrombocytopenia. He was managed with oral hydration and supportive care, although outpatient laboratory values were unavailable. He lived in a poorly ventilated environment with high ambient temperatures and had prolonged daytime heat exposure due to his occupation.

On arrival, his blood pressure was 120/70 mmHg, heart rate 110 beats/min, respiratory rate 22 breaths/min, and temperature 41°C. Neurological examination revealed a Glasgow Coma Scale score of 3/15, with equal and reactive pupils, generalized hypotonia, preserved deep tendon reflexes, and bilateral flexor plantar responses. His skin was hot and dry. Given the marked hyperpyrexia, environmental heat exposure, and neurological collapse, environmental heat stroke was considered an important initial differential diagnosis.

**Figure 1:**
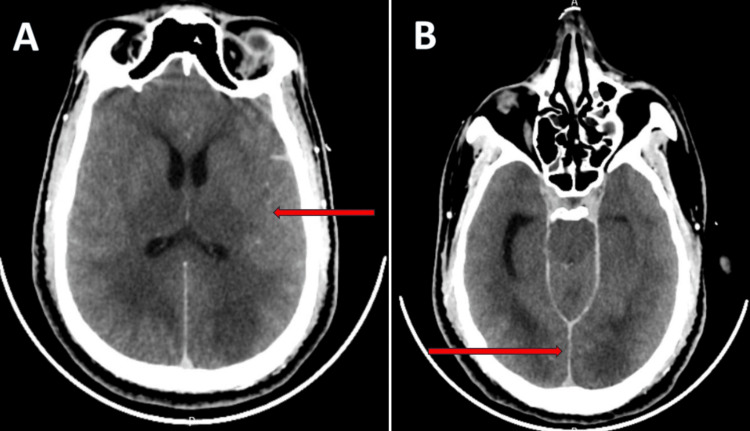
Non-contrast CT brain demonstrating diffuse cerebral edema and subarachnoid hemorrhage (A) Axial CT image shows diffuse cerebral edema with loss of gray-white matter differentiation and effacement of cortical sulci (arrow). (B) Axial CT image demonstrates hyperdensity within the interhemispheric fissure consistent with subarachnoid hemorrhage (arrow). CT: computed tomography

Shortly after arrival, he developed respiratory arrest requiring emergency endotracheal intubation and transfer to the intensive care unit. Active cooling was initiated with intravenously administered cooled normal saline (approximately 1-2 liters during initial resuscitation) and external cooling measures, including ice packs and surface cooling techniques, resulting in a body temperature of 39°C. Non-contrast computed tomography of the brain demonstrated subarachnoid hemorrhage and diffuse cerebral edema (Figure 1). Intravenous mannitol was administered at a dose of 1 g/kg with monitoring of serum sodium and osmolality.

Initial laboratory investigations revealed hemoglobin 15.4 g/dL, hematocrit 41.3%, white blood cell count 3.47 × 10⁹/L, and platelet count 70 × 10⁹/L. Coagulation parameters were abnormal at presentation, with a prothrombin time of 18.5 seconds and an activated partial thromboplastin time of 56 seconds. D-dimer was elevated at 1.1 mg/L. Liver enzymes were elevated (AST 554 U/L, ALT 399 U/L). Initial serum lactate was 1.6 mmol/L and progressively increased over 48 hours (to 1.8, 2.2, 2.7, and 4.6 mmol/L), indicating worsening tissue hypoperfusion. Malaria screening was negative, and blood cultures were obtained and subsequently reported negative. Other differential diagnoses, including viral encephalitis and other hemorrhagic febrile illnesses, were considered but were less consistent with the evolving clinical and laboratory findings.

Within two hours of ICU admission, he developed hypotension (89/50 mmHg) with tachycardia, raising concern for transition into the plasma leakage phase of dengue shock syndrome. Fluid resuscitation was initiated according to World Health Organization dengue guidelines [1] using isotonic crystalloid boluses of 10-20 mL/kg administered over 15-30 minutes, with close reassessment of hemodynamic status, urine output, and serial hematocrit measurements.

Due to persistent hypotension, vasopressor support was initiated in accordance with Surviving Sepsis Campaign recommendations. Norepinephrine was started at 0.05 µg/kg/min and titrated up to 0.5 µg/kg/min to maintain a mean arterial pressure ≥65 mmHg. Vasopressin at a fixed dose of 0.03 units/min was subsequently added for refractory shock.

Over the next 24-48 hours, the patient developed progressive hemoconcentration, worsening thrombocytopenia, mucosal bleeding, and further derangement of coagulation parameters, along with marked transaminitis, acute kidney injury with oliguria (urine output <0.5 mL/kg/h), hypernatremia, and worsening metabolic acidosis with rising lactate levels. These findings were consistent with evolving coagulopathy suggestive of disseminated intravascular coagulation, although complete diagnostic parameters, including fibrinogen levels and formal scoring systems, were unavailable (Table 1).

**Table 1:** Serial laboratory trends demonstrating hemoconcentration, coagulopathy, and multiorgan dysfunction over 48 hours. Values are shown at admission and at subsequent 12-hour intervals up to 48 hours. Hb: hemoglobin, HCT: hematocrit, WBC: white blood cell count, Na⁺: sodium, K⁺: potassium, ALT: alanine aminotransferase, AST: aspartate aminotransferase, Cr: creatinine, BUN: blood urea nitrogen, PT: prothrombin time, APTT: activated partial thromboplastin time, PaO₂: partial pressure of oxygen, PaCO₂: partial pressure of carbon dioxide

Parameters	Admission	12 hours	24 hours	36 hours	48 hours	Reference range
							Hb (g/dL)	15.4	17.1	17.6	19.5	19.2	10.6-13.5
HCT	41.3	46.5	50.6	56.2	55.9	36-46
WBC (×10⁹/L)	3.47	6.4	8.9	11.49	12	4-11.5
Platelet (×10⁹/L)	70	72	36	26	28	150-450
Total Bilirubin (mg/dL)	0.8	1.1	1.2	1.3	1.4	0.2-1.3
ALT (U/L)	399	419	456	686	689	<35
AST (U/L)	554	724	886	1325	1992	14-36
Cr (mg/dL)	1.4	1.3	2.2	5	5.7	0.52-1.04
BUN (mg/dL)	6.1	12	12.1	23.4	36	7.0-17.0
Na⁺ (mmol/L)	144	163	172	180	184	135-145
K⁺ (mmol/L)	2.9	4,6	2.6	2.4	3.6	3.5-5.5
D-Dimer (ug/mL FEU)	1.1	1.2	3.85	3.88	3.9	<0.5
PT	18.5	16.1	16.8	20.4	20	11.0-16.0
APTT	56	55.6	58.1	73	68	27.9-41.9
Lactate(mmol/L)	1.6	1.8	2.2	2.7	4.6	0.4-2.0
pH	7.301	7.211	7.239	7.132	7.11	7.35-7.45
PaO2(mmHg)	133	170	101	66.3	62	83-108
PaCO2(mmHg)	45.2	55.5	46.3	48.8	43	32-48

Management included transfusion of fresh-packed red cells, platelet concentrates, and fresh-frozen plasma; invasive mechanical ventilation; vasopressor support; and careful fluid titration guided by clinical response and hematocrit trends. Despite these interventions, he remained in refractory shock with progressive lactate elevation and died approximately 48 hours after ICU admission. The clinical course was most consistent with severe dengue complicated by plasma leakage, coagulopathy, and progressive multiorgan dysfunction.

## Discussion

This case illustrates a fulminant presentation of expanded dengue syndrome with early neurological involvement and rapid progression to multiorgan dysfunction. The patient presented with seizures and coma, with neuroimaging demonstrating subarachnoid hemorrhage and cerebral edema. Neurological complications of dengue are uncommon but increasingly recognized, with proposed mechanisms including direct viral effects, immune-mediated injury, metabolic disturbances, and hemorrhagic complications [3-5].

In this case, intracranial hemorrhage occurred early in the disease course, in the presence of thrombocytopenia and abnormal coagulation parameters. While endothelial dysfunction and vascular injury may have contributed, the exact mechanism cannot be definitively established. This highlights that intracranial bleeding can occur even in the absence of profoundly deranged coagulation parameters and should be considered in dengue patients presenting with neurological symptoms [6,8].

An important clinical feature was the initial presentation mimicking environmental heat stroke. Severe hyperpyrexia, altered consciousness, and significant heat exposure created a strong diagnostic overlap. However, the subsequent rise in hematocrit, progressive thrombocytopenia, mucosal bleeding, and development of shock were consistent with severe dengue.

The hematocrit increases from 41% to 56% over the first 24-36 hours, reflecting significant plasma leakage, a hallmark of dengue shock syndrome [1]. This transition occurred around day 4-5 of illness, corresponding to the critical phase of dengue, during which vascular permeability typically increases.

The progressive rise in lactate from 1.6 to 4.6 mmol/L further supports worsening tissue hypoperfusion and shock severity. The subsequent development of coagulopathy, hepatic dysfunction, renal failure, and metabolic acidosis reflects advanced multiorgan dysfunction. Dengue-associated coagulopathy is multifactorial, involving endothelial activation, hepatic dysfunction, and consumption of clotting factors [6-8]. Persistent shock despite fluid resuscitation, vasopressor support, neurocritical care, and blood product replacement was most consistent with uncontrolled plasma leakage and progressive multiorgan dysfunction.

This case highlights an important clinical learning point: severe dengue may closely mimic heat stroke in its early presentation, particularly in individuals with significant heat exposure, and failure to recognize dengue-related plasma leakage may delay appropriate management.

## Conclusions

This case describes a severe and fatal presentation of expanded dengue syndrome initially mimicking environmental heat stroke. The clinical course, including rising hematocrit, progressive thrombocytopenia, coagulopathy, increasing lactate levels, and refractory shock, was consistent with severe dengue progressing to dengue shock syndrome and multiorgan dysfunction. Persistent shock despite aggressive supportive therapy was most consistent with uncontrolled plasma leakage and progressive organ failure.

In dengue-endemic tropical settings, clinicians should maintain a high index of suspicion for severe dengue in patients presenting with hyperpyrexia, neurological symptoms, or shock, even when features resemble heat stroke. Early recognition, close hematocrit monitoring, and prompt escalation of care are essential to improve outcomes.
